# Do single people want to date a cancer survivor? A vignette study

**DOI:** 10.1371/journal.pone.0194277

**Published:** 2018-03-22

**Authors:** Marrit Annika Tuinman, Vicky Lehmann, Mariët Hagedoorn

**Affiliations:** Department of Health Psychology, University of Groningen, University Medical Center Groningen, Groningen, the Netherlands; Tulane University, UNITED STATES

## Abstract

**Objective:**

Qualitative studies indicated that cancer survivors may be worried about finding a partner in the future, but whether this concern is warranted is unknown. We examined single people´s interest in dating a cancer survivor, how they perceive survivors’ traits, and their preferences about the timing of disclosing a cancer history.

**Methods:**

In three experimental vignette studies, dating website members (*n* = 324) and college students (*n* = 138 and *n* = 131) were randomly assigned to a vignette of a person with or without a history of cancer (experiment 1 & 2), or a cancer survivor beyond or during active follow-up (experiment 3). Respondents rated their interest in dating this fictive person, this person’s traits, and indicated their preferences about the timing of disclosure. ANOVAs with main and interaction effects of condition, gender, and relationship history were conducted, partial eta squared and Cohen’s *d* were used to estimate the magnitude of effects. Correlations were used to investigate relationships between interest in a date and assessment of traits.

**Results:**

Cancer survivors’ traits were assessed more positively, but interest to date them did not differ from healthy vignettes for both men and women. However, widowed respondents were much less interested in a date with a cancer survivor, and women showed less interest in a cancer survivor during active follow-up relative to survivors beyond follow-up. Most respondents wanted to hear about the cancer diagnosis after a few dates, hardly anyone wanted to hear about this before the first date (2% - 5%).

**Conclusion and implications for cancer survivors:**

Cancer survivors do not have to expect any more problems in finding a date than people without a cancer history, and can wait a few dates before disclosing. Survivors dating widowed people and survivors in active follow-up could expect more hesitant reactions and should disclose earlier.

## Introduction

Finding a romantic partner is a central goal in life for most people and essential for well-being [1,2]. Especially when dealing with a stressful life event as cancer, having a partner can be advantageous: Partnered people on active cancer treatment adapt better both physically and psychologically as compared to those without a partner [3–13]. However, knowledge about establishing a new relationship following cancer is lacking. This is surprising, given the fact that around 40% of young adults, and 15% of middle aged people who have been diagnosed with cancer are single [14]. In addition, several studies showed that cancer survivors are less often married or partnered as compared to healthy peers [15–17]. As a result, there is a growing population of single cancer survivors who will be faced with finding a new partner after they completed their treatment.

Qualitative studies revealed several issues that cancer survivors experience when they are looking for a partner. For example, survivors reported feeling insecure and different [18], feeling negative about their bodies [19] or less sexually desirable, while they can also worry about late effects like infertility, and whether this could be a deal breaker for future partners [18,20]. Survivors specifically worry about negative reactions from potential partners toward their appearance and fear rejections, making the disclosure of their cancer history more difficult [21–24].

However, dating and initiating a relationship depends on two people. Investigating the attitude of healthy individuals towards cancer survivors as potential romantic partners is essential in order to understand how relationship formation might be affected by cancer. How individuals think about starting a relationship with someone who has had cancer is unknown, but we do know that people have a tendency to react negatively to others who are ill or disabled. Many people are distressed by thinking or actually meeting a cancer patient, and as a result avoid contact, leaving patients feeling socially isolated [25–28]. Despite the current improvements in treatment and therefore survival, many people unaffected by cancer still have negative and discriminating attitudes toward cancer, with even a majority of 70% agreeing with a statement that cancer patients are not able to contribute to society, and around a quarter indicating that they would avoid working with people who have cancer [29]. Another study found similar negative sentiments and distancing in the general population, especially among people who have had fewer personal experience with cancer [30]. These negative and avoiding responses are found to be even greater with respect to cancer types that others deem as being a result of one’s own bad health behavior, such as lung cancer [31]. However, attitudes about cancer may have improved more recently, due to media presentations of people treated for cancer as being courageous [32,33]. This may have affected people’s current views of survivors and their attitudes toward dating them. Therefore, we designed three vignette studies to examine whether single people would want to date a cancer survivor, and explored people’s views about positive and negative traits of these vignettes. We embedded cancer information in a subtle way into these vignettes (rather than directly asking people about their interest in dating cancer survivors) to account for social desirability.

Interest in dating a cancer survivor may also differ by gender. Although men place relatively more value on physical attractiveness and women on social status [34,35], they both value health, dependability, stability, education and intelligence in a long-term mate [36,37]. However, women are usually the more choosy sex, meaning that they are less open for romantic contact than men and more critical when they search for a partner. From the perspective of evolutionary psychology, women have evolved to be the more choosier sex, where they look for traits in a partner that increase the chance of protection, provision and investment in potential children. This is a result of women having much higher parental investment (being pregnant, nursing. and rearing children) [34]. Thus, a mistake in mate choice, and consequently having a child with a wrong mate, is costlier in all aspects for women than it is for men. Studies show how women, all around the world, value dependability, stability, education and intelligence in a long- term mate more than men do [37]. When situations arise where one needs to choose between different traits of a potential partner, researchers found that women listed nearly four times as many desired traits of a potential partner then men did, women also remained more selective than men in situations where there were few partners/mates to choose from [38]. Also, men are more willing to compromise on the traits they ideally desire in a potential partner than women, meaning that men would accept mates that do not meet (all of) their requirements more than women would, for example regarding health [39].

We hypothesize that (1) single people will be less interested in dating a cancer survivor than a similar partner without a cancer history. We further hypothesize that women will be less interested in romantic contacts than men, and (2) that this difference will be more pronounced if the potential partner is a cancer survivor. To test our hypotheses, we conducted three experimental vignette studies among members of a national Dutch dating website (experiment 1) and university students (experiment 2 and 3), using fictive profiles of a potential dating partner.

### Experiment 1: Interest in a cancer survivor among members of a dating website

Many singles look for a potential date or partner online ever since dating websites became available [40]. In the USA, roughly 20% of heterosexual couples said to have established their first contact online [41]. In the Netherlands, a more recent study showed that 43% of singles look for a date or partner online [42]. To study reactions to a cancer survivor among single people who are actively looking for a date or partner, we chose to recruit participants who were members of a dating website. Participants were presented with a written profile of either a cancer survivor or someone without a cancer history (while other aspects were kept identical). Besides testing our two main hypotheses, we tested whether desire to date a survivor depends on participants’ relationship history. Members of a dating website have different relationship histories, ranging from never-married, to divorced, or widowed. It has been found that a change from being married to unmarried (either divorced or widowed) is related to lower well-being, as compared to those who were never married [2,43,44]. It might be that people who have experienced the loss of a partner are less interested in making contact with a cancer survivor, as cancer invokes ideas about death and potentially losing a partner again [26]. Therefore we hypothesize (3) that people who are divorced or widowed will show less interest in dating a cancer survivor than single people who did not experience divorce or death of a spouse.

#### Method

**Participants & Design:** An invitation to participate in a study evaluating dating profiles was advertised by three Dutch dating websites, indicating the study was done by the University of Groningen. One website advertised the link to the online survey in their bi-weekly email newsletter sent to members, the other two posted the ad on their blog or website.

The online survey started with informing participants about the voluntary and anonymous character of this study done by the University of Groningen. They were asked to indicate their consent by ticking a box and could then proceed to the questionnaire (not ticking the box would let them exit the questionnaire). Basic demographic questions about gender, education level, marital status (single, divorced, widowed, in a relationship), sexual orientation, and geographic region were asked. Respondents were then randomly assigned to the experimental vignettes. The dating profile was introduced as following: “*A lot of people in the Netherlands are interested in coming into contact with other singles through dating websites*. *How people present themselves in their profile can have a big impact on the number of responses they get*. *Next*, *we will show you a transcript of a profile text*, *without a picture (due to privacy reasons)*. *The person in the profile is selected by the computer*: *you both live in the same region and are in the same age category*.” A gender neutral scenario was written similar to profiles presented on dating websites. The description entailed work (teacher), hobbies (playing tennis and mountain biking), and personality characteristics (social, spontaneous, active, funny and sometimes stubborn). This description was the same for the two conditions. In the middle of the profile, the manipulation was presented. In the cancer profile, it was stated “*Since I completed treatment for cancer two years ago*, *I am grateful for being alive and I enjoy the simple things in life more*: *such as going out for dinner*, *laughing together*, *drinking a glass of wine*, *but I also enjoy going on a holiday or short trip*”. In the healthy profile, this part was “*Since I almost lost my best friend in a car crash two years ago*, *… [identical text]*”. The Ethical Committee of the psychology department of the University of Groningen, the Netherlands, approved this study (ppo-015-082).

In total, 424 participants participated, but 41 (10%) were excluded due to technical errors and 59 (15%) were excluded because they were in a committed relationship. This resulted in a final sample of 324 respondents, of which 186 (44%) were randomized to rate a healthy profile and 238 (56%) rated a cancer profile (see Table 1, column experiment 1). No group differences in age (F(1, 303) = 0.9, p = .340), gender (*χ*^*2*^ (1) = 0.39, p = .533) or relationship history (*χ*^*2*^ (2) = 0.24, p = .887) were found between respondents in the healthy vs. the cancer condition in experiment 1. Of the total sample, 92% had experience with cancer within their family (i.e., a parent (30%), grandparent (30%), sibling (11%), aunt/ uncle (37%)), a friend (49%), and/or a romantic partner (10.5%); note that respondents could indicate several categories if applicable.

**Table 1:** Descriptive statistics of respondents in all experiments.

	Experiment 1	Experiment 2	Experiment 3
	Dating website	Students	Students
	N = 324	N = 138	N = 131
	n (%)	n (%)	n (%)
**Condition**			
Healthy profile	186 (44%)	66 (48%)	
Beyond follow-up	238 (56%)	72 (52%)	54 (45%)
Active follow-up			67 (55%)
**Gender**			
Men	132 (41%)	45 (33%)	37 (28%)
Women	192 (59%)	93 (67%)	94 (72%)
**Relationship status**			
Single, never dated	-	43 (32%)	49 (37%)
Single, dated before	-	55 (39%)	34 (26%)
Single, relationship before	-	40 (29%)	48 (37%)
Single, never married	198 (61%)	-	-
Divorced	101 (31%)	-	-
Widowed	25 (8%)	-	-
**Same sex preference**	22 (7%)	4 (3%)	7 (5%)
**Age**			
M (SD)	37.7 (15.2)	19.3 (1.4)	19.2 (1.4)
**range**	17–78	16–25	18–26
**Divorced (M, SD)**	51.7 (9.1)	-	-
**Never-married (M, SD)**	29.5 (10.4)	-	-
**Widowed (M, SD)**	63.1 (9.1)	-	-
Educational level*			
**M (SD)**	6.1 (1.4)	7	7
**range**	2–9	7	7

* Educational level was measured with categories ranging from primary school (1) to a Master’s degree (9)

**Variables & measures:** After reading the vignette, respondents indicated on a visual analog scale of 0–10 (i.e. no—yes) whether they would date this person (i.e. dating interest). Next, they indicated on an 11-point Likert-type scale (0 = not at all, 10 = very much) how they rated the fictive person on being sympathetic, interesting, athletic, attractive, healthy, friendly, and funny. We report scores for each of these characteristics separately.

#### Results

**Hypothesis 1 & 2: less interest in cancer survivors, especially for women and Hypothesis 3: less interest among divorced and widowed respondents:** A univariate general linear model with interest in a date as dependent variable and condition (healthy vs. cancer), gender, and relationship status (divorced, never married, widowed) and the interaction terms as fixed factors (F(11, 312) = 3.2, p < .001, η^2^ = .102) showed a main effect for condition (F(1, 312) = 4.6; p = .033, η^2^ = .014, *d* = 0.11) and gender (F(1, 312) = 5.9, p = .015, η^2^ = .019, *d* = 0.44), but no main effect for relationship status (F(2, 312) = 0.5, p = .585, η^2^ = .003). Men and participants in the healthy condition were more interested in a date, yet the interaction between condition and gender was not significant (F(1,312) = 0.4, p = .515, η^2^ = .001). However, the interaction between condition and relationship status was significant (F(2, 312) = 4.1, *p* = .017, η^2^ = .026). Simple effects analysis showed that only widowed respondents had significantly lower interest in dating a cancer survivor than divorced singles (mean difference = 1.87, SE = 7.9, p = .024; *d* = 0.82), and never married singles (mean difference = 1.49, SE = 7.7, p = .054; *d* = 0.67). In the healthy condition, interest did not differ by relationship status (S1 Fig and Table 2).

**Table 2:** Interest in dating in all experiments.

	Members dating website experiment 1						Students experiment 2			Students experiment 3		
	Total	Men	Women	Single	Divorced	Widowed	Total	Men	Women	Total	Men	Women
	M (SD)	M (SD)	M (SD)	M (SD)	M (SD)	M (SD)	M (SD)	M (SD)	M (SD)	M (SD)	M (SD)	M (SD)
**Total ratings**	2.5 (2.8) n = 324	3.3 (2.9) n = 132	2.0 (2.6) n = 192				4.1 (2.5) n = 138	5.3 (2.5) n = 46	3.6 (2.3) n = 92	4.6 (2.4) n = 121	4.0 (2.2) n = 35	4.9 (2.4) n = 86
**Healthy**	2.7 (2.9)	3.3 (2.9) n = 61	2.3 (2.9) n = 82	2.4 (2.6) n = 89	2.9 (3.3) n = 44	4.1 (3.7) n = 10	3.9 (2.4)	4.9 (2.6) n = 23	3.6 (2.3) n = 43			
**Cancer**	2.4 (2.7)	3.3 (2.9) n = 71	1.8 (2.4) n = 110	2.6 (2.8) n = 109	2.9 (2.7) n = 57	1.1 (1.6)^a^ n = 15	4.3 (2.5)	5.8 (2.4) n = 23	3.7 (2.3) n = 49			
**Beyond follow-up**										5.3 (2.4)	4.1 (1.9) n = 15	5.8 (2.4)^b^ n = 39
**Active follow-up**										4.0 (2.2)	3.9 (2.5) n = 20	4.1 (2.1) n = 47

Mean (Standard Deviation), all scales ranged from 0–10;

^a^ only widowed respondents showed a conditional difference in interest

^b^ only women showed a conditional difference in interest.

**Assessment of traits:** General linear models with the traits as dependent variable and condition and gender as fixed factors showed that cancer and healthy profiles were rated as similar on the presented traits, except for health (F(1, 298) = 13.0, p < .001, η^2^ = .042). The profile in the cancer condition was rated as less healthy than the healthy condition profile (M = 6.3 vs. 7.2; *d* = .58), without main or interaction effects of gender. The healthy trait was positively, albeit weakly to moderately correlated with the likelihood to date this person in both conditions, meaning that the more healthy they assessed the presented person, the more interested they were in dating them (Table 3). Overall, the traits correlated in a similar way and strength with interest in a date between the healthy and the cancer condition, except for athletic.

**Table 3:** Correlations between interest in a date and assessed traits.

	Experiment 1		Experiment 2		Experiment 3	
	Healthy	Cancer	Healthy	Cancer	Beyond follow-up	Active follow-up
	r (p)	r (p)	r (p)	r (p)	r (p)	r (p)
**Interesting**	**.51** (.001)	**.44** (.001)	**.59** (.001)	**.44** (.001)	**.44** (.001)	**.46** (.001)
Independent*			**.33** (.01)	**.33** (.004)	-.03 (.86)	**.39** (.001)
Insecure*			.20 (.11)	.10 (.38)	**.44** (.001)	-.07 (.60)
**Friendly**	**.32** (.001)	**.25** (.001)	.22 (.08)	**.29** (.02)	**.31** (.02)	**.45** (.001)
**Healthy**	**.18** (.03)	**.25** (.001)	**.31** (.01)	**.52** (.001)	**.41** (.002)	**.29** (.01)
Experienced*			.21 (.09)	**.29** (.01)	.01 (.95)	**.45** (.001)
Needy*			-.002 (.99)	.17 (.14)	**.51** (.001)	.11 (.39)
**Funny**	**.40** (.001)	**.30** (.001)	**.36** (.003)	**.25** (.03)	**.49** (.001)	**.53** (.001)
Brave*			**.37** (.002)	.19 (.11)	**.34** (.01)	**.28** (.02)
Strong*			**.48** (.001)	**.31** (.01)	-.25 (.07)	**.43** (.001)
Sympathetic*	**.29** (.001)	**.18** (.02)				
Athletic*	.16 (.06)	**.18** (.02)				
Attractive*	**.45** (.001)	**.35** (.001)				

* Empty cells due to using an adapted list of traits after experiment 1.

#### Discussion

Among members of a dating website, interest in dating a cancer survivor was lower than interest in a comparable person without a cancer history. Although this finding supported our first hypothesis, this difference was negligible in effect size. Women were found to be less interested in a date than men (supporting our second hypothesis), but this gender difference did not depend on whether the potential partner had cancer or not. Our third hypothesis, that divorced and widowed people would be less interested in dating a cancer survivor was only supported for widowed people. Widowed singles showed almost no interest in dating a cancer survivor, a huge difference to their interest in a healthy partner/date (*d* = 3.8). We expected to find the same difference for divorced people, but that was not the case. It may be that specifically losing a partner to death makes widowed people reluctant to dating someone who has had cancer (and might die). They may have also lost their previous partner to cancer and may want to avoid going through such an experience again. Therefore, it can be expected that having had cancer is relevant for older survivors looking for a new partner, as they are more likely to encounter someone who is widowed. Nevertheless, the examined subsample of widowed participants was also very small (n = 25), and caution is needed in extrapolating our findings to the entire group of widowed people looking for a potential partner or date.

Although this experiment was done in a highly relevant context (i.e. single people actively looking for dates), some aspects needed improvement. We noticed that respondents were not very likely to see themselves dating the presented person (i.e. numerous respondents filled in a 0 percent interest). We speculate that a missing picture may be a vital reason for this overall low interest. Looks are an extremely important first trigger of interest [45]. In order to improve upon this potential pitfall, we decided to include a profile picture in our second experiment.

### Experiment 2: Interest in a cancer survivor among young adults

Because members of a dating website are a diverse population when it comes to age and previous relationship status, we aimed to test our hypotheses in a more homogeneous group of young adult single people. In early adulthood, other reasons for dating may prevail in comparison to older adulthood. While older adults might aim for marriage, young adults tend to aim for short-term partners and less relationship involvement, and desire someone who is similar to them [46]. During this phase in life, it could be that a cancer history implies that the other person is less similar (at a young age, few people have been confronted with such a serious life event), resulting in less interest. A previous experiment in a student sample showed that respondents saw themselves as less similar to someone with cancer, than a patient with a sprained ankle [47]. Also, at a young age, physical appearance and sexual traits such as passion and sexual responsiveness are considered more important in a sexual or romantic partner than at an older age [48,49]. To account for this, we first assessed interest in the person (before learning about the cancer history) to examine whether the disclosure of a cancer history would decrease this initial interest. We then asked participants when they would like to learn about a cancer history from a dating partner. Qualitative studies reported that young adult cancer survivors sometimes struggle with when and how to tell a potential new partner about their cancer history [22,50], but study findings from healthy partners perspectives are missing.

#### Method

**Participants & design:** First year medicine students (N = 355) were invited to participate in an online survey during a lecture and through their digital study guide. Students did not receive credits for their participation, but were informed that this study was part of their colloquium and that they would be informed about the results in an upcoming lecture. In total, 225 students (RR = 63%) participated. Students indicated their relationship status as either single or in an exclusive relationship, of which 87 (38%) were in an exclusive relationship and therefore excluded for further analyses. This resulted in a remaining sample of 138 respondents (see Table 1, column experiment 2). No group differences in age (F(1,137) = 1.3, p = .254, η^2^ = .009), gender (*χ*^*2*^ (1) = 0.18, p = .676) or relationship history (*χ*^*2*^ (2) = 6.0, p = .050) were found between respondents in the healthy vs. the cancer condition in experiment 2.

The voluntary and anonymous character of the study was explained online and participants were informed that by proceeding with the questionnaire, they indicated their consent with participation. Participants were asked to indicate basic demographic information before they were randomized to the healthy or cancer condition (see Table 1, column experiment 2). They were presented with a description and picture of a fictive fellow student. Gender of the fictive student was matched by sexual orientation and pictures showed a close-up with a happy facial expression (used from the Radboud Faces Database [51]. The male and female pictures were selected based on age (comparable to the age of first year students) and attractive looks. The student was described as a second year student that passed all exams, had a part-time job in a theatre, lived in student housing, sometimes visited his/her parents, was single but would like to be in a relationship. After answering several questions, respondents were presented with part 2 of the profile, presenting either a story for the healthy condition (lost best friend to cancer three years ago) vs. the cancer condition (had cancer him or herself three years ago). The Ethical Committee of the psychology department of the University of Groningen, the Netherlands, approved of this study (ppo-015-082).

**Variables and measures:** After reading the first part of the scenario, respondents rated on a visual analog scale (no (1)—yes (10)) whether they would be interested in a date with this person (initial interest). After being randomized, they again rated interest in a date, followed by ratings of the presented person on 10 traits (i.e., interesting, independent, insecure, friendly, healthy, experienced, needy, funny, brave, strong) on a scale of 1–10 (not at all—very much). Finally, all participants (including those in the healthy condition) were asked when they would like to learn about a cancer history of a potential romantic partner (i.e., before the first date, at the first date, after a few dates, when the relationship was considered exclusive).

#### Results

**Hypothesis 1 & 2: less interest in cancer profile, especially in women:** Univariate general linear model with condition (healthy vs. cancer) and gender as fixed factors and interest in a date as measured in part 2 (i.e., after cancer disclosure) as dependent variable was performed. We accounted for initial interest as measured in part 1 and entered it as covariate (initial interest and interest after second part of description correlated strongly, *r* = .86, *p <* .001). The overall model was significant (F(4,134) = 97.1; *p <* .001, η^2^ = .744), with initial interest (F(1,134) = 351.9, *p <* .001, η^2^ = .724) explaining almost all of the variance of dating interest; while main effects of gender (F(1, 134) = 0.46, p = .497, η^2^ = .003) and condition (F(1, 134) = 1.12, p = .292, η^2^ = .008), and their interaction (F(1, 134) = 2.69, p = .103, η^2^ = .020) were not significant (see Table 2 for means). Accordingly, a similar analysis without initial interest as covariate changed the overall results (F(3,135) = 3.4, p = .020, η^2^ = .070), with no significant main effect for condition (F(1, 135) = 1.01, p = .316, η^2^ = .007) or the interaction effect (F(1, 135) = 0.2, p = .211, η^2^ = .012). However, without accounting for initial interest, a significant yet small effect of gender was found, where men (M = 5.5, SD = .2.4) reported greater interest in a date overall than women (M = 4.3, SD = 2.4; F(1,135) = 8.1, p = .005, η^2^ = .057).

**Assessment of traits:** General linear models with condition and gender as fixed factors and the traits as dependent variable showed differences in three of the seven traits. Respondents in the cancer condition assessed the presented profile as more interesting (M = 6.2, *SD* = 1.6) than respondents in the healthy condition (M = 5.6, *SD* = 2.0; *F*(1,135) = 5.6, *p* = .020; *d* = 0.33*)*, as well as more brave (M = 6.9; SD = 1.7 vs M = 6.1; SD = 1.9, F(1,135) = 6.7, *p* = .011; *d* = 0.39) and stronger (M = 6.7; SD = 1.7 vs M = 5.9; SD = 1.8; F(1,135) = 7.7, *p* = .006; *d* = 0.46). No significant effects were found for gender or the interaction of condition and gender. Correlations between the assessment of traits and interest in a date varied somewhat between conditions (Table 3). In both conditions, respondents were more interested in a date when they assessed the person as being more interesting, independent, healthy, funny, and strong. However, interest of respondents in the healthy condition was also related to them seeing the person as more brave, whereas this was not the case in the cancer condition. Interest of respondents in the cancer condition was related to their assessment of the other person as being friendly and experienced, whereas this was not the case in the healthy condition.

**Best time for disclosure:** In the total group, the vast majority (n = 105, 76%) wanted to learn about a cancer history after a few dates, 7% (n = 10) at the first date, 1% (*n* = 2) before the first date, and 1% (n = 1) when agreeing to have an exclusive relationship, 20 respondents indicated ‘other’ (15%). These preferences differed between conditions (*χ*^*2*^*(4)* = 12.2, *p* = .016), with respondents in the cancer condition answering more often that they would like to learn about this at the first date (11% vs. 3%), and none of them (0% vs. 3%) indicating that they would like to hear about this when deciding to have an exclusive relationship. To account for an empty cell in the cancer condition, we excluded the answering category `when exclusive´ and reran the analysis, with the result remaining significant (χ^2^(3) = 10.9, *p* = .012).

#### Discussion

Young adult single students were as interested in dating another student who was treated for cancer a few years earlier than someone without such an illness history. Their initial interest in the presented person was the strongest factor associated with their final interest in a date, not the illness history. This suggests that interest in dating a person does not change once someone hears about a cancer history (as also indicated by their strong correlation). In addition, these students also attributed positive traits to the cancer survivor such as being brave and strong, while not assessing the survivor as less healthy (which was the case with dating website members). These results indicate that some of the worries young cancer survivors have expressed in qualitative studies with respect to dating are unwarranted. It may be that the effect of media coverage of having cancer is shifting from something to be feared [52] to something that can be conquered and beaten [32,53]. These students, on average 19 years old, have probably seen many campaigns and posters providing them with success stories of survivors, while their real-life experience with cancer may be more limited. When young adult survivors start dating a new romantic partner, they can expect that others would prefer them to disclose this early on, specifically after a few dates.

This second experiment was designed more rigorously than the first one, as we added information on initial interest in a date, to account for basic liking of the person without knowing their illness history. Also, we added a profile picture of a person with a similar age to the description. However, both experiments presented a survivor who was beyond the treatment phase. Thus, interest in dating might be different if individuals are faced with potentially dating a cancer patient who is closer to diagnosis and still being regularly checked by their medical providers. Therefore, we built upon experiment 2 and designed experiment 3 which we presented to students in the next academic year, and varied conditions based upon illness statuses: being beyond the follow-up phase versus having regular follow-up appointments to monitor disease activity.

### Experiment 3: Interest in a date and phase of disease follow-up

In the years following end of active treatment, people treated for cancer remain in follow-up. This means they are regularly checked for their health, and for possible tumor activity to detect metastases or relapse. Some survivors use medication to lower the chance of recurrence. It is possible that fear or distancing from a serious illness is larger when confronted with someone who is still having regular check-ups at the hospital, and therefore may be seen as not yet fully cured. Also, the illness still plays a role in daily life shortly after treatment completion. Therefore, we hypothesized that students would be less interested in dating someone who has had cancer and is still under close medical monitoring as compared to a survivor who no longer regularly visits the hospital for check-ups related to the treatment of cancer.

#### Method

**Participants and design:** The method and procedure was similar to experiment 2, only the vignettes were adapted to differentiate between two phases of cancer survivorship. The online survey was presented to first year medicine students in the year following experiment 2. Procedures were kept exactly the same and the two conditions were (a) a fellow student with a cancer history, who “*never has hospital check-ups anymore*, *because the cancer is gone*” (beyond follow-up) versus (b) a fellow student who is a cancer survivor who “… *still regularly visits the hospital to monitor whether the cancer is really gone*” (active follow-up). In total, 226 of 350 first year medical students (RR = 65%) started the questionnaire, of which 37 (16%) discontinued participation shortly after start. Of the remaining 189 participants, 68 (36%) were in a committed relationship and excluded for further analyses. This resulted in a sample of 131 respondents, of which ten left the survey before randomization (see Table 1, column experiment 3). Participants in the beyond follow-up condition were on average 19.5 years old (SD = 1.6) and participants in the active follow-up condition were 18.9 years (SD = 0.95) which was significantly different (F(1, 119) = 4.8, p = .02), while the distribution of gender (*χ*^*2*^ (1) = 062, p = .803) and relationship histories (*χ*^*2*^(2) = .883, p = .643) were similar between respondents of both conditions.

#### Results

**Hypothesis 1 & 2: less interest in survivor in active follow-up, especially in women:** Univariate general linear model with condition (beyond follow-up vs. active follow-up) and gender as fixed factors and interest in a date as measured in part 2 as dependent variable was performed. We entered initial interest (as measured in part 1, before illness disclosure) as covariate. The overall model was significant (F(4,116) = 40.8; *p* < .001, η^2^ = .584), with significant main effects of initial interest (F(1,116) = 129.8, *p* < .001, η^2^ = .528), condition (F(1,116) = 6.1, *p* = .015, η^2^ = .050), and gender (F(1,116) = 10.8, *p* = .001, η^2^ = .085). The interaction term of condition and gender (F(1,116) = 5.1, *p* = .025, η^2^ = .042) was also significant, but explained hardly any variance of interest in a date (4%). Simple effects analysis showed no differences in interest between conditions for men, but women showed less interest in a date in the active follow-up condition relative to the beyond follow-up condition (mean difference = 1.5; SE = .34; *p* = .001; *d* = 0.75). It also appeared that in the beyond follow-up condition, women showed more interest in a date than men, which was contradictory to our hypothesis (Table 2). Analysis was repeated without initial interest as covariate (F(3,117) = 5.3, p = .002, η^2^ = .119), which also showed significant, but small main effects for condition (F(1,117) = 4.4, p = .037, η^2^ = .037) and gender (F(1,117) = 4.2, p = .043, η^2^ = .035) but not for the interaction effect (F(1,117) = 2.5, p = .113, η^2^ = .021). Explained variance of the overall model without the covariate was much smaller (58% versus 12%), indicating that in this sample the initial liking of the presented person was more important for being interested in a date than a history of illness, just as in experiment 2.

**Assessment of traits:** General linear model with condition and gender as fixed factors and the traits as dependent variables showed that survivors in the active follow-up condition were seen as more independent, less insecure, more friendly, less healthy, more experienced, and stronger than survivors in the beyond follow-up condition (Table 4). These differences were moderated by gender for independent, insecure and strong, indicating that only women judged survivors in the active follow-up condition as more independent (mean difference = -3.0, SE = 0.42, *p* < .001), less insecure (mean difference = 2.9, SE = 0.39), *p* < .001), and stronger (mean difference = -4.9, SE = 0.42, *p* < .001), than women in the beyond follow-up condition. Men did not assess these traits differently between conditions. Also, even though no main effect for condition was found for needy and funny, women assessed the survivor in the beyond follow-up condition as more needy relative to men (mean difference = 1.7, SE = 0.58, p = .003) but also more funny than men (mean difference = 1.5, SE = .49, p < .001). All traits correlated with interest in a date, which varied between conditions (Table 3). In the beyond follow-up condition, participants were more interested in a date when they assessed the person as being more insecure and needy, whereas this was not the case in the active follow-up condition (nor in the other conditions of experiment 2). Interest of participants in the active follow-up condition was positively related to their assessment of the person as independent, experienced, and strong, whereas this was not the case in the other condition.

**Table 4:** Assessment of traits in experiment 3.

Trait	Beyond follow-up		Active follow-up		Test		Effect size
	M	(SD)	M	(SD)			
**Interesting**							
Total	6.4	(2.0)	6.3	(1.9)	Condition	F(1, 117) = 0.4	*d* = 0.1
Men	5.4	(1.8)	6.2	(2.3)	Gender	F(1, 117) = 14.6	
Women	6.9	(1.9)	6.3	(1.6)			
					Interaction	F(1, 117) = 11.1	
**Independent**							
Total	4.4	(2.3)	6.8	(1.9)	Condition	F(1, 117) = 21.8***	*d* = 1.1
Men	6.3	(2.2)	7.0	(2.2)	Gender	F(1, 117) = 13.5***	
Women	3.7	(1.9)	6.7	(1.8)			
					Interaction	F(1, 117) = 8.8**	
**Insecure**							
Total	6.1	(2.2)	3.9	(1.7)	Condition	F(1, 117) = 19.4***	*d* = 1.1
Men	4.0	(2.1)	3.8	(2.1)	Gender	F(1, 117) = 17.8***	
Women	6.9	(1.7)	3.9	(1.6)			
					Interaction	F(1, 117) = 13.8***	
**Friendly**							
Total	5.2	(1.6)	6.7	(1.6)	Condition	F(1, 117) = 16.4***	*d* = 0.9
Men	5.5	(1.6)	6.5	(1.7)			
Women	5.1	(1.6)	6.7	(1.6)	Gender	F(1, 117) = .03	
					Interaction	F(1, 117) = 0.8	
**Healthy**							
Total	5.7	(1.9)	4.6	(1.8)	Condition	F(1, 117) = 5.4*	*d* = 0.6
Men	5.3	(1.7)	4.9	(1.8)	Gender	F(1, 117) = .06	
Women	5.8	(2.0)	4.6	(1.8)			
					Interaction	F(1, 117) = 1.1	
**Experienced**							
Total	4.6	(2.1)	6.2	(2.3)	Condition	F(1, 117) = 8.5**	*d* = 0.7
Men	5.9	(1.9)	6.3	(2.4)	Gender	F(1, 117) = 4.9*	
Women	4.1	(1.9)	6.2	(2.3)			
					Interaction	F(1, 117) = 3.3	
**Needy**							
Total	4.9	(1.8)	4.2	(2.1)	Condition	F(1, 117) = 0.9	*d* = 0.4
Men	3.7	(1.6)	4.2	(2.3)	Gender	F(1, 117) = 4.8*	
Women	5.4	(1.6)	4.2	(2.0)			
					Interaction	F(1, 117) = 5.4*	
**Funny**							
Total	6.3	(1.7)	5.5	(1.6)	Condition	F(1, 117) = 2.6	*d* = 0.5
Men	5.3	(1.5)	5.5	(1.8)	Gender	F(1, 117) = 4.9*	
Women	6.7	(1.6)	5.5	(1.6)			
					Interaction	F(1, 117) = 5.4*	
**Brave**							
Total	6.6	(1.7)	6.9	(2.3)	Condition	F(1, 117) = 1.1	*d* = 0.1
Men	6.1	(2.2)	6.7	(2.3)	Gender	F(1, 117) = 1.3	
Women	6.7	(1.5)	7.0	(2.2)			
					Interaction	F(1, 117) = 0.1	
**Strong**							
Total	2.9	(2.5)	6.6	(2.2)	Condition	F(1, 117) = 45.7***	*d* = 1.6
Men	6.3	(2.3)	6.8	(2.2)	Gender	F(1, 117) = 38.4***	
Women	1.7	(1.0)	6.6	(2.3)			
					Interaction	F(1, 117) = 31.1***	

* *p* < .05,

** *p* < .01,

*** *p* < .001.

Effect size Cohen´s *d* for condition.

**Best time to disclose:** Preferences regarding the best time to disclose were somewhat different from the second experiment. Again, few people preferred a disclosure before the first date (5%). However, a much larger group of participants wanted to hear about the cancer history at the first date (37% vs. 7% in experiment 2), fewer people after a few dates (48% vs. 76% in experiment 2), and 4% when agreeing to have an exclusive relationship. Responses differed between conditions (*χ*^*2*^(4) = 48.9, *p* < .001), with the respondents in the beyond follow-up condition answering more often that they would like to learn this at the first date (64% vs. 15%) and less after a few dates (17% vs. 73%) than in the active follow-up condition. In the active follow-up condition, no one wanted to hear about this when agreeing to be exclusive (0% vs. 9% in the beyond follow-up condition). To account for an empty cell in the active follow-up condition, we excluded the answering category `when exclusive´ and reran the analysis, with the result remaining significant (χ^2^(3) = 72.4, p < .001).

#### Discussion

When students were presented with a cancer survivor as a potential dating partner, responses differed by disease trajectory (i.e., beyond vs. active follow-up), but not when presented with a healthy person versus a cancer survivor (as shown in experiment 2). It seems that a more recent health issue (and for example dealing with more rigorous surveillance and uncertainty about relapses) does result in some hesitation to go on a date with potential partners with a cancer history. In contrast to the first two experiments, where we found only few of our hypotheses were supported, this experiment showed some support. Similar to experiment 2, initial interest in the presented person was the strongest factor in being interested in a date. However, women were less interested in a date with someone who was in active follow-up, while men’s interest did not differ between conditions. This finding underscores women’s more critical selection of potential partners, as they reported less interest when a current health issue was present. However, this is striking given that women assessed survivors in active follow-up as more positive (e.g., stronger, less insecure) than the survivors who were beyond follow-up. Thus, even though single women saw them as having more desirable traits, they were less interested in a date. It has to be noted though that when comparing men and women in the survivor beyond follow-up condition, women were more interested in a date than men, which was unexpected. We can only speculate about underlying factors of this finding, but we did see some differences in traits that uniquely correlated with interest between conditions. Interest of students in someone who was having regular check-ups (active follow-up) was related to more positive traits like independent, healthy, experienced and strong which is a more expected finding (an active student, going on with life despite health insecurities). Whereas for the beyond follow-up condition, student´s interest was positively related to their assessment of the survivor as insecure and needy, while this was not the case in the active follow-up condition. In contrast to all other conditions in the first experiments, women had a higher interest than men to date someone beyond follow-up. It may be that especially women’s interest was triggered by seeing this person as more needy and insecure, or wanting to get into contact with that person on a date. It might also be an artifact due to the rather small sample of male participants in this experiment. Unfortunately, due to the small number of men, correlations between conditions could not be purposefully explored by gender as well. In addition, and different from the second experiment, most respondents wanted to learn about the cancer history at the first date as opposed to hearing about it after a couple of dates. However, for both experiments, the more serious health condition (no vs. a cancer history, regular check-ups vs. beyond follow-up) was related to wishing an earlier disclosure. Early disclosure seems more warranted when survivors are closer to diagnosis.

## Summary and overall discussion

In sum, these three experiments showed that ever-single and divorced people are as likely to be interested in a date with a cancer survivor as with someone without a cancer history, unless they are still in active follow-up. Survivors were also judged as more positively than other people (e.g., brave), as were survivors who went for regular medical check-ups in comparison to survivors who were beyond follow-up for their treatment. Nevertheless, these positive traits did not make them more attractive as a potential partner. It might be that people are afraid of possible relapses, other long-term side effects, and the foresight of regular hospital visits. This is emphasized by the finding that widowed single people hardly showed any interest in a date with a cancer survivor. They probably did not want a chance to experience losing a loved one again.

The advantages of our online experiments were in the design. Vignette studies are especially useful when it is difficult to experimentally manipulate sensitive topics [54]. In the dating-setting, people tend to uphold their own dating standards and preferences more so at a distance than face-to-face. Any critical attitudes or socially undesirable thoughts would have had more room in our design because the people who were judged were not physically present. This is supported by a study on ideal partner preferences which showed that these preferences are mostly upheld in an abstract context, such as reading about a potential partner online, and are less important when there is face-to-face contact [45]. However, more studies should focus on previously partnered and middle-aged people. This could shed more light on their motivation to date or not to date someone who has had cancer. Additionally, our student sample just started medical school and they may be more understanding and less anxious when meeting people who were or are facing a serious illness. Future studies should include a more representative sample of young adult single people. Our limited subsamples of widowed (25 participated) and divorced respondents can only represent a starting point for future studies. These groups may be specifically important for future research as cancer is more common among older adults. Their preferences and dating behaviors when finding a new partner (with or without cancer) might be of particular importance for future research and the clinical practice. Nevertheless, they may not be very active on dating websites and other forms of recruitment for studies may be explored. In line with this, our experiments need replication as the latter two included young adults with a high educational level and overrepresented women. Young people are not very likely to have personal experiences with peers who have had cancer, which may positively color their ideas about what it means to be a cancer survivor and its impact. Furthermore, we think more research attention is needed to gain insight into actually establishing and maintaining romantic relationships after cancer, beyond the phase of getting on a first date.

Our experiments only touched upon initial liking and did not include any form of communication or how people would actually respond when survivors disclose their cancer history. It is advised for survivors to keep in mind that most people wanted this disclosure after some contact and initial interest arose, and not immediately at the first date or before meeting. Actual responses might differ between chatting online and meeting face-to-face and talking about the cancer experience, which actually also gives the other person the opportunity to ask and clarify questions about the cancer experience [55]. It is possible that hearing about the actual challenges of having had cancer will invoke more fear or distancing in the dating partner. The same holds true for the survivors as well: they could also be more or less interested after receiving actual responses from others when they disclose. In line with such reasoning, partnered cancer survivors who receive loving, caring, and understanding responses from their spouse, show less depressive symptoms [56]. Receiving these positive responses when disclosing to a potential partner will probably have an important impact on whether the survivor wants to continue dating or not. Current cancer advocate campaigns now focus on successes of survivors, calling them heroes and emphasizing strength [32,33]. This may be reflected in the positive trait assessments of survivors we found. However, the reality of having survived cancer is more confronting. We believe future studies on finding a partner after cancer should focus on more sensitive topics (such as lasting infertility or changed appearance), and the way survivors and their dating partners handle conversations about this.

## Implications

Cancer survivors who completed treatment can expect the same success in finding a date than people without a cancer history, and can wait until after a few dates to disclose. Survivors who are still being regularly checked for disease activity, and somewhat older survivors who potentially date widowed people, could expect more hesitant reactions. They could also disclose their experience with cancer earlier, but not before the first in-person meeting. Reactions toward actual disclosure and risks for potential discontinuation of dating need to be explored in cancer survivors. Future studies should focus on how survivors could best start a conversation about having had cancer when they are looking for a romantic partner.

## Supporting information

S1 Dataset (7Z)

S1 Fig Interaction effect between relationship history and health condition. Note: The y-axis displays estimated mean values of interest in a date.
(TIF)
