# Supplementary figures and images for: Do single people want to date a cancer survivor? A vignette study
Source: PLoS One. 2018 Mar 22;13(3):e0194277. doi: 10.1371/journal.pone.0194277 (PMC5863988; doi:10.1371/journal.pone.0194277)

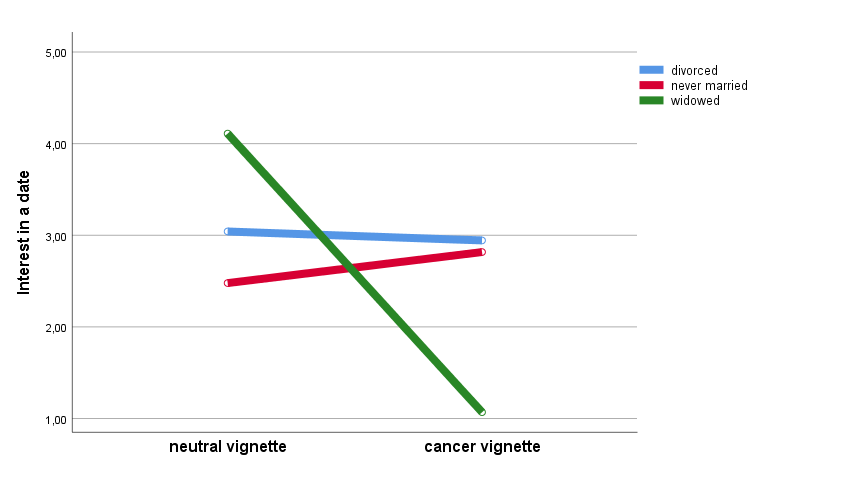

Supplement: S1 Fig — Note: The y-axis displays estimated mean values of interest in a date. (TIF) [file pone.0194277.s002.tif]
